# Fluorine labelling of therapeutic human tolerogenic dendritic cells for ^19^F-magnetic resonance imaging

**DOI:** 10.3389/fimmu.2022.988667

**Published:** 2022-10-03

**Authors:** Fiona Cooke, Mary Neal, Matthew J. Wood, I. Jolanda M. de Vries, Amy E. Anderson, Julie Diboll, Arthur G. Pratt, James Stanway, Ioana Nicorescu, Nicholas Moyse, Dawn Hiles, David Caulfield, Anne M. Dickinson, Andrew M. Blamire, Pete Thelwall, John D. Isaacs, Catharien M. U. Hilkens

**Affiliations:** ^1^ Translational & Clinical Research Institute, Newcastle University, Newcastle upon Tyne, United Kingdom; ^2^ Research into Inflammatory Arthritis Centre Versus Arthritis (RACE), Newcastle upon Tyne, United Kingdom; ^3^ Newcastle Magnetic Resonance Centre, Newcastle University, Newcastle upon Tyne, United Kingdom; ^4^ Division of Rheumatology, Rush University Medical Centre, Chicago, IL, United States; ^5^ Department of Tumour Immunology, Radboudumc, Radboud Institute for Molecular Life Sciences, Nijmegen, Netherlands; ^6^ Musculoskeletal Unit, Newcastle upon Tyne Hospitals NHS Foundation Trust, Newcastle upon Tyne, United Kingdom; ^7^ Newcastle Advanced Therapies, Newcastle upon Tyne NHS Hospitals Foundation Trust, Newcastle upon Tyne, United Kingdom

**Keywords:** tolerogenic dendritic cells, ^19^F, cell tracking, ^111^Indium, MRI, rheumatoid arthritis

## Abstract

Tolerogenic dendritic cell (tolDC) therapies aim to restore self-tolerance in patients suffering from autoimmune diseases. Phase 1 clinical trials with tolDC have shown the feasibility and safety of this approach, but have also highlighted a lack of understanding of their distribution *in vivo*. Fluorine-19 magnetic resonance imaging (^19^F-MRI) promises an attractive cell tracking method because it allows for detection of ^19^F-labelled cells in a non-invasive and longitudinal manner. Here, we tested the suitability of nanoparticles containing ^19^F (^19^F-NP) for labelling of therapeutic human tolDC for detection by ^19^F-MRI. We found that tolDC readily endocytosed ^19^F-NP with acceptable effects on cell viability and yield. The MRI signal-to-noise ratios obtained are more than sufficient for detection of the administered tolDC dose (10 million cells) at the injection site *in vivo*, depending on the tissue depth and the rate of cell dispersal. Importantly, ^19^F-NP labelling did not revert tolDC into immunogenic DC, as confirmed by their low expression of typical mature DC surface markers (CD83, CD86), low secretion of pro-inflammatory IL-12p70, and low capacity to induce IFN-γ in allogeneic CD4^+^ T cells. In addition, the capacity of tolDC to secrete anti-inflammatory IL-10 was not diminished by ^19^F-NP labelling. We conclude that ^19^F-NP is a suitable imaging agent for tolDC. With currently available technologies, this imaging approach does not yet approach the sensitivity required to detect small numbers of migrating cells, but could have important utility for determining the accuracy of injecting tolDC into the desired target tissue and their efflux rate.

## Introduction

Rheumatoid arthritis (RA) is a debilitating inflammatory autoimmune disease, which is caused by a loss of immune tolerance to self-antigens. Current treatments target inflammatory pathways in a non-antigen-specific manner, leading to general immunosuppression and increased susceptibility to infection. Although these treatments can alleviate symptoms, they need to be administered long-term and do not restore the underlying cause of the pathology.

A current focus is to develop treatments that reinstate immune tolerance to self, potentially providing long-lasting clinical effects without affecting protective immunity to pathogens. Tolerogenic dendritic cells (tolDC) offer a promising approach to achieve this. They act by presenting antigen to T cells in the context of pro-tolerogenic signals, thereby restoring the dysregulated immune response through induction of regulatory T cells, T cell hyporesponsiveness (anergy), as well as suppression of pro-inflammatory cytokine production by self-reactive effector T cells (1–3).

We have developed a tolDC therapy for RA, where tolDC are manufactured *ex vivo* from monocytes of the patient’s own peripheral blood, loaded with relevant autoantigens, and injected back into the patient (4). We have established a robust method to generate stable tolDC by treating immature monocyte-derived DC with dexamethasone, the active form of vitamin D3, and a TLR4 ligand. We have previously shown that these tolDC have potent immunoregulatory activity *in vitro*, including the induction of type I regulatory T (Tr1) cells (5–9) and that equivalent murine tolDC suppress symptoms of inflammatory arthritis in animal models (10, 11). We recently completed a phase 1 clinical trial showing that injection of these autologous tolDC into an inflamed joint is safe (12). The rationale for this route of administration was that it would provide the opportunity for tolDC to act locally, through suppression of pathogenic self-reactive T cells in the synovium, as well as systemically, through the induction of regulatory T cells in secondary lymphoid tissue. The latter would require migration of tolDC from the joint. However, although reduced synovitis was observed in the joints of patients treated with the highest tolDC dose, no evidence of systemic immune modulation was found. Moreover, a follow-on study using ^111^Indium-labelled tolDC in one RA patient showed that these cells failed to migrate out of the joint (**Supplementary Figure 1**), which could explain the lack of systemic effects of tolDC treatment.

A critical question that needs addressing is the optimal injection route for tolDC to target autoantigen-specific T cells and reinstate immune tolerance. Clinical trials to date have employed different injection routes, including intra-dermal, -lymph nodal, -venous, -peritoneal, and -articular (13) but these studies have not directly compared the immunomodulatory efficacy of tolDC administered *via* these different routes. Moreover, the behaviour and distribution of tolDC after injection into a patient remains an understudied area, precluding a deeper understanding of where they exert their actions, therefore limiting the ability to enhance the clinical efficacy and applicability of these cell therapies.

The use of magnetic resonance imaging (MRI) is currently considered to be a comprehensive non-invasive method for tracking of DC pre-labelled with an imaging agent containing fluorine (^19^F) nuclei for detection by ^19^F-MRI (14). ^19^F-MRI benefits from observing the ^19^F nucleus which is highly MR sensitive compared to most other MR-visible nuclei, and there is negligible endogenous ^19^F signal *in vivo*. In the cancer immunotherapy field, studies have been carried out to optimise labelling of immunogenic DC with fluorinated compounds, ensuring that the process did not negatively affect DC phenotype, function, and survival, whilst maintaining their detectability by ^19^F-MRI *in vivo* in mice (15–18) and humans (19). However, one study noted that labelling of DC with ^19^F-containing particles did enhance maturation of DC (17) and although this would not necessarily be detrimental for DC therapeutics that are designed to act in an immunogenic manner, it could potentially have a negative impact on tolerogenic DC therapies.

Here, we explored the use of ^19^F-nanoparticles (^19^F-NP) for labelling of tolDC and their detection by ^19^F-MRI. These ^19^F-NP are constructed from biodegradable polymers (poly(d,l-lactide-co-glycolide) - PLGA) that encapsulate perfluorcarbon as well as indocyanine green (ICG), thus allowing for detection by both ^19^F-MRI and flow cytometry. Importantly, they are available as a Good Manufacture Practice (GMP) compliant product (Cenya Imaging), a prerequisite for their application in clinical trials, and have been reported to yield the highest cellular ^19^F labelling so far (20). As our aim was to use ^19^F-labelled tolDC in our imminent clinical trial, we tested the i) compatibility of these ^19^F-NP with our therapeutic tolDC using a set of assays that we are employing as quality control criteria, and ii) detectability by ^19^F-MRI.

## Materials and methods

The minimum information about tolerogenic antigen presenting cells (MITAP) checklist was used for this paper (21).

### Blood

Peripheral blood samples and Leukocyte Reduction System cones (NHS Blood and Transfusion Service, NHS, UK) were obtained from healthy human volunteers. Samples were collected with informed consent and following a favourable ethical opinion from The Animal Welfare and Ethical Review Body (AWERB), Newcastle University.

### ^19^F nanoparticle synthesis

Clinical-grade PLGA nanoparticles with entrapped perfluorocarbon in combination with gadoteridol (Prohance™; Bracco Imaging Europe) and a fluorescent dye, IC-Green (Akorn Inc., IL, USA) were prepared by Cenya Imaging (the Netherlands) using a mini-emulsion formulation method (20). All non-sterile solutions were filtered through an 0.22 mm filter. PLGA (100 mg, Resomer RG 502 H, lactide:glycolide molar ratio 48:52 to 52:48; Evonic Industry, Germany) was dissolved in dichloromethane (3 ml) and mixed rapidly with PFCE (900 μl, Exfluor Inc., TX, USA), after which IC-Green (1 mg) and Prohance™ (1780 µl) were added. This mixture was sonicated for 8 seconds at an amplitude of 40% at ≤21°C using a digital sonicator from Branson Ultrasonics (Connecticut, USA). The mixture was then added to an aqueous solution of poly(vinyl alcohol) (2.5% m/v) and emulsified for 3 minutes under sonication at 90% amplitude at ≤21°C. The solvent was evaporated overnight at room temperature under stirring in open class I glass flacons. Particles were collected by centrifugation (35 min, 13304 RCF, 4 °C) and washed 3 times with cooled water-for-injection (WFI). After these washing steps, the particle pellets were homogenized on a programmable rotator. Subsequently, 3 washing steps with cooled WFI were performed before the particle pellets were homogenized again, pooled and resuspended in WFI and lyophilized, generating a powder with a light green tinge. ^19^F content ranges from 5.8 × 10^18^ to 1.3 × 10^19 19^F atoms/mg (determined with trifluoroacetic acid as internal reference). Gadoteridol content was measured using inductively coupled plasma mass spectrometry (ICP MS); the particles typically contain 0.5 μg/mg.

### Tolerogenic and mature DC

Peripheral blood mononuclear cells (PBMC) were isolated by gradient density centrifugation using Lymphoprep (Alere). Monocytes were further separated from PBMC by positive selection using CD14^+^ magnetic microbeads and a MACS LS column (Miltenyi) according to manufacturer’s instructions. Monocytes were seeded into 24-well culture plates (Corning) at 0.5 x 10^6^/ml (total of 1 ml per well) in GMP DC medium (CellGenix) containing IL-4 and GM-CSF (both at 50 ng/ml; Immunotools) and cultured for 7 days at 37°C, 5% CO_2_. On day 3, IL-4 and GM-CSF were refreshed at 50 ng/ml final concentration, and half of the medium was replaced with fresh GMP DC medium. To generate tolDC, dexamethasone (Sigma) was added at 10^-8^ M on days 3 and 6 of culture, and 10^-10^ M 1,25-dihydroxyvitamin D3 (either Calcitriol from Tocris or Decostriol from Mibo) and 1 µg/ml of the TLR4 agonist monophosphoryl A (MPLA; Avanti) on day 6. To generate mature DC (matDC), cells were treated with 1 µg/ml MPLA (Avanti) on day 6 of culture only. In addition, on day 6 tolDC were labelled with ^19^F-NP (1.1, 2.2 or 4.4 mg/ml) or left unloaded as a control. DC were harvested on day 7, resuspended in HBSS + 1% FCS in a 30 ml universal tube (Starlab) and placed on ice for further processing (see sections below). Viability and cell numbers were determined by trypan blue (Sigma).

### Cryopreservation and thawing

DC were cryopreserved in pre-cooled GMP DC medium (CellGenix) supplemented with 40% Human Serum Albumin (HSA)(Biotest) and 10% DMSO (Sigma) in a cryovial (Corning). Cryovials were immediately placed in a cool cell in a -80°C freezer for 24 hours before transfer into a storage box. Cryovials were stored short-term (less than one month) in a -80°C freezer or in liquid nitrogen long-term (more than one month). Cells were thawed in a 37°C water bath until ice crystals dissolved then transferred to an appropriate centrifuge tube where at least 5 ml of pre-warmed HBSS (Lonza) with 10% FCS (Biosera) was slowly added. The tubes were then topped up with HBSS with 10% FCS and washed in HBSS with 10% FCS by centrifugation twice at 400 xg for 8 minutes at room temperature before being counted and viability assessed by trypan blue (Sigma).

### Flow cytometry

As part of the formal release criteria of therapeutic tolDC, we set up a flow cytometry panel to define the identity, contamination and viability of the cells. The following mAbs were incubated with cells, on ice in the dark for 30 minutes: CD11c (Biolegend), CD83 (Becton Dickinson), CD86 (Becton Dickinson), HLA-DR (Biolegend), TLR2 (Thermo Fisher), CD3 (BD), CD19 (BD), CD41 (Thermo Fisher), CD56 (BD) and CD66b (Biolegend), as well as relevant isotype-matched controls (see **Table 1 in Supplementary Data** for full details on mAbs; CE marked mAbs were used where possible). Zombie Aqua (Biolegend) was added to cells and incubated in the dark for 15 minutes to assess cell viability. Cells were fixed in 1% formaldehyde (TAAB Labs) and stored in the dark at 4°C for a maximum of 7 days. Cells were analysed on a Canto II or Fortessa flow cytometer (Becton Dickinson). Compensation was set up using compensation beads (for mAbs; Becton Dickinson) or using a mixture of stained and unstained cells (for Zombie Aqua) or unloaded and cells loaded with ^19^F-NP. Results were analysed by FCS Express RUO software (Denovo Software) or FlowJo software (FlowJo). Cells were gated by excluding debris and selecting the single cells. Cell viability and expression of surface markers (median fluorescent intensity (MFI) and percentage positive cells) was then calculated from this population.

### ^19^F-MRI of tolDC phantoms

Phantoms for MRI analysis were produced by heating 1% low melting point agarose in a water bath or microwave until all agarose had dissolved. Agarose was then briefly allowed to cool before layering 750 µl into a 1.5 ml microcentrifuge tube on ice. The agarose in the tube was allowed to set before adding a layer of DC resuspended in 200µl HBSS + 1% FCS. 100 µl of agarose was added on top of this and an additional 300 µl of agarose added again once the previous layer had set. Once set the tubes were stored at 4°C, protected from light until MRI was performed. MR imaging was performed on a Philips Achieva 3.0 Tesla (T) scanner (Philips Healthcare, Best, NL), equipped with a 20 cm diameter ^19^F-tuned surface coil (PulseTeq Ltd., UK) placed level with the scanner bed and loaded with 2 x 500 mL bags of 0.9% saline solution. The agar phantom was positioned upright, with DC suspension fixed 3 cm above the coil centre. ^19^F images were acquired with a 2D steady state free precession (SSFP) sequence with the following acquisition parameters: field of view = 160 x 160 mm^2^, voxel size = 10 x 10 mm^2^, slice thickness = 50 mm, echo time/repetition time = 2.5/5.3 ms, flip angle = 60°, bandwidth = 370 Hz/pixel, signal averages = 16,384, scan duration = 23 minutes. Analysis was performed in Matlab (MathWorks, Natick MA, USA) using the complex image data. Mean ^19^F signal ( $\bar{S}$ ) was measured from a manually drawn region of interest (ROI) around the signal locus. Noise mean ( $\bar{N}$ ) and standard deviation (*σN* ) were calculated from a 5 x 5 cm^2^ ROI placed in a location devoid of ^19^F signal. SNR was calculated as ( $\bar{S}\;$ – $\bar{N}$ )/*σN*

### Potency assay

As part of the formal release criteria of therapeutic tolDC, we optimised a ‘potency assay’ as an *in-vitro* surrogate for *in-vivo* activity to confirm that tolDC lacked the ability to induce IFNγ production by allogeneic cells. Allogenic PBMC from 4 different donors (10^5^ cells/well for each donor) were thawed (see above) and co-cultured with each of the DC populations (mature DC and/or tolDC; 10^4^ cells/well each) in a 96 well plate (Corning) in 200 µl of GMP DC medium (CellGenix) supplemented with 4% HSA (Biotest) in duplicate. After 4 days the supernatants from duplicates were combined, divided into aliquots and stored at -80°C for assessment of IFNγ by ELISA.

### ELISA

Day 7 DC culture supernatants were assessed for IL-10 and IL-12p70 and potency assay supernatants were assessed for IFNγ cytokine levels by ELISA. EIA plates (Corning) were coated with capture antibody (Becton Dickinson) and left overnight at 4°C. The next day plates were washed (PBS + 0.1% Tween-20) and block (PBS + 1% BSA) was added for at least 1 hour at room temperature. During the 1-hour incubation, samples and cytokine standards (Becton Dickinson) were thawed and diluted. The plates were then washed and samples and cytokine standards added to the plate and left to incubate overnight at 4°C. On the third day, plates were washed 4 times and detection antibody (Becton Dickinson) added. Plates were incubated at room temperature for 1 hour then washed and ExtrAvidin (Sigma) added and left for 30 minutes at room temperature. This was followed by 5 washes and the addition of OPD (Sigma) to develop colour and 3 M H_2_SO_4_ after at least 20 minutes to stop the reaction. Plates were read at 490 nm by a plate reader (Labtech). Microsoft Excel (Microsoft Corporation) was used to plot a concentration curve and calculate cytokine concentrations of the samples.

### Transmission electron microscopy (TEM)

Day 7 DC were washed twice in glutaraldehyde and resuspended in glutaraldehyde for at least 1 hour to fix. Cells were then enrobed in 4% agar before further fixation and dehydration followed by processing to resin. A Hitachi TEM was used to image cells.

### Confocal microscopy

13 mm round glass slips (VWR) were prepared in 24 well plates by sterilising in 70% ethanol before monocyte seeding. PBMC separated monocytes were seeded in these 24 well plates and cultured for 7 days as described above. On day 7 DC on the glass cover slips (VWR) were washed, stained with DAPI (Thermo Scientific) and mounted with antifade mounting medium (Invitrogen) on a glass slide (Corning). Slides were left to dry for at least 24 hours in a dark area before transfer to a fridge. Imaging was performed on Zeiss LSM800 AiryScan confocal microscope using APC (ex651-em660) and DAPI (ex353-em465) channels with Plan-Apochromat 40x/1.30 Oil M27 objective. Image analysis was performed on Zen 2 software.

### Statistics

Statistical software in GraphPad prism 9 was used to produce and analyse graphs (GraphPad). Microsoft Excel was used to input, organise and calculate averages for flow cytometry, ELISA and Potency Assay data. For experiments where paired sample data were produced, Wilcoxon test was used to assess statistical significance. In the case of unpaired samples Kruskal-Wallis or Friedman test was used. Spearman’s Rank Correlation Coefficient was used to determine the correlation and significance of ^19^F-NP concentration and viability. A one-tailed binomial test was used to determine IL-12 ELISA detection significance between tolDC and matDC or ^19^F-tolDC and matDC.

## Results

### Labelling of tolDC with 19F-NP nanoparticles

**Figure 1:**
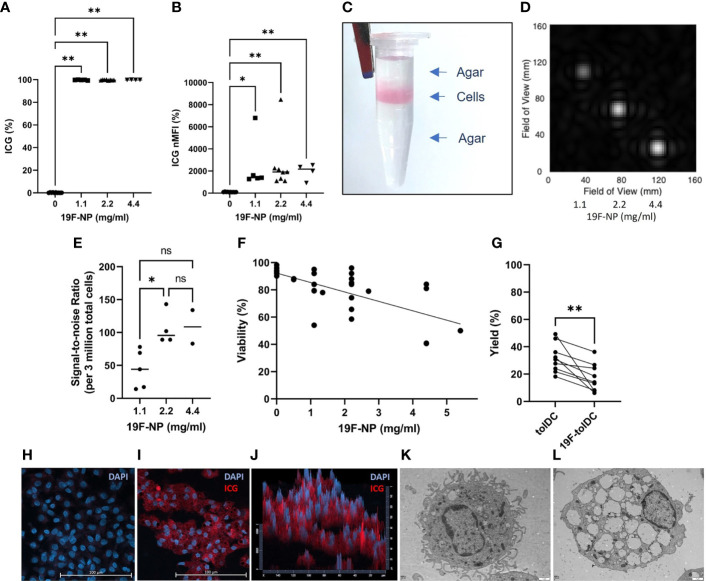
Uptake of ^19^F-NP by tolDC. ^19^F-NP (0-5.4 mg/ml) was added to tolDC for the last 20-24 hours of culture, after which cells were harvested, washed and counted. Uptake of ^19^F-NP was determined by flow cytometry, data are shown as **(A)** percentage of cells positive for the ICG label and **(B)** as normalised median fluorescent intensity (nMFI) by dividing the MFI obtained for ^19^F-tolDC by the MFI obtained for unlabelled tolDC then multiplied by 100. ^19^F-tolDC were incorporated into an agar phantom **(C)** for detection by MRI at 3 concentrations of ^19^F incubation. Left to right; 1.1 mg/ml, 2.2 mg/ml and 4.4 mg/ml; scan time 23 mins 0 secs **(D, E)** data are depicted as the signal-to-noise ratio per 3 million tolDC. **(F)** Viability of tolDC was determined by flow cytometry using Zombie Aqua and **(G)** tolDC yield calculated as a percentage of the monocytes seeded on day 0. tolDC were cultured on glass slips and incubated with and without 2.2 mg/ml 19F PLGA for 20-24 hours. The glass slips were washed and stained with DAPI (blue) to indicate nuclei. tolDC on the slips were imaged using confocal microscopy. Nuclei (blue) were detected in unlabelled tolDC **(H)** whilst nuclei (blue) and ^19^F-NP (red) were detected in the ^19^F-tolDC **(I)**. The ^19^F-NP is localised to the cytoplasm of the cells and is present throughout the cytoplasm as visualised in the pseudo 3D plot **(J)**. TEM images of tolDC **(K)** and ^19^F-tolDC **(L)** show distinct endocytic structures present in the cytoplasm of the 19F-tolDC. Graphs **(A, B, E)** show individual values with the median as a line (*p≤ 0.05, **p ≤ 0.01). Graph **(F)** displays individual values with a line of regression. Spearman’s Rank Correlation Coefficient was performed on the samples and r found to be -0.6870 with a p-value<0.0001. Graph **(G)** displays paired values connected by a line (**p≤ 0.01). NS, not significant.

The optimal concentration of ^19^F-NP nanoparticles for tolDC labelling was determined by assessing uptake and detection of these particles, as well as the effect of the labelling process on cell viability. ^19^F-NP nanoparticles are dually loaded with perfluorcarbon and ICG, allowing for detection by both MRI and fluorescence-based detection technologies. Initial flow cytometric analyses showed that labelling of tolDC with ^19^F-NP consistently resulted in nearly 100% of the cells taking up the nanoparticles (**Figure 1A**), irrespective of the ^19^F-NP concentration. The normalised median fluorescence intensity (MFI) values suggested some variability in the level of uptake, but again this was independent of the ^19^F-NP concentration used, with all labelling conditions showing a significantly higher normalised MFI compared to unlabelled tolDC, but not to each other (**Figure 1B**). **Figure 1C** shows an agar phantom containing ^19^F-tolDC for measurement of detectability by ^19^F-MRI. The field of view for a typical MRI scan (**Figure 1D**) demonstrates the intensity of signal-to-noise ratio (SNR) between increasing concentrations of ^19^F-NP detected. Higher concentration of ^19^F-NP in the tolDC medium resulted in higher ^19^F -MR signal. A statistically significant difference in ^19^F SNR is seen at a ^19^F-NP concentration of 2.2mg/ml compared to the 1.1 mg/ml concentration (**Figure 1E**). Viability of ^19^F-tolDC was variable but suggested a significantly negative correlation with the dose (p<0.05) (**Figure 1F**). Although the yield of the tolDC product was significantly reduced by labelling with ^19^F-NP (**Figure 1G**), the reduction was not prohibitive for the production of sufficient numbers of therapeutic tolDC and was therefore deemed acceptable. Uptake of ^19^F-NP by tolDC was confirmed by confocal microscopy (**Figures 1H–J**) and electron microscopy (**Figures 1K, L**), showing the presence of the ICG label or the merged ^19^F-NP PLGA membranes within the cytoplasm of the cells, respectively. Taken together, the data show that tolDC efficiently ingest ^19^F-NP and that the labelled cells are detectable by MRI. The 2.2 mg/ml concentration was taken forward for further work as it allowed for optimal detectability by MRI with acceptable tolDC viability and yield.

### ^19^F-tolDC surface marker expression

**Figure 2:**
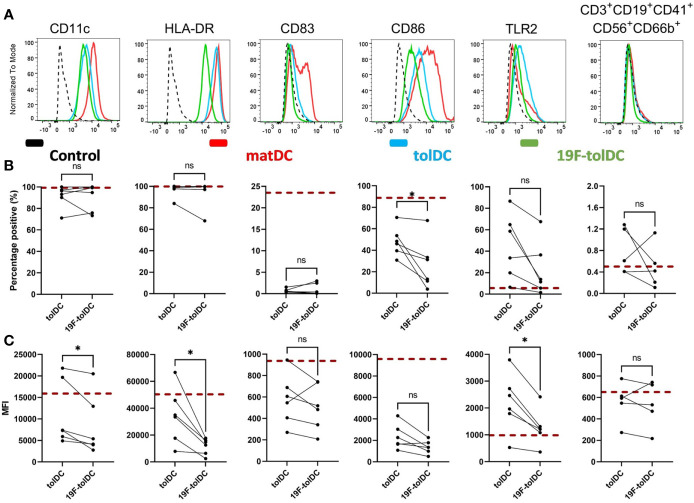
Cell surface marker expression of tolDC and ^19^F-tolDC. ^19^F-NP (2.2 mg/ml) was added to tolDC for the last 20-24 hours of culture, after which cells were harvested, washed and expression of tolDC QC markers was determined by flow cytometry. **(A)** Histograms (normalised to mode) of isotype-matched control (dashed line), mature DC (red line), tolDC (blue line) and ^19^F-tolDC (green line). The graphs display expressing the QC surface markers by tolDC and ^19^F-tolDC as percentages **(B)** and MFI **(C)** Red dashed line indicates median values of the matDC control. Graphs show 6 repeats, connecting line indicates paired samples (*p≤ 0.05). NS, not significant.

^19^F-tolDC surface marker expression was determined with a set of validated QC markers, used for our previous (12) and imminent tolDC clinical trial in RA patients. For formal QC purposes only the percentage of cells positive will be taken into consideration, but for completeness we have also presented MFI data (**Figure 2**). CD11c and HLA-DR were used for identification of cells as myeloid antigen-presenting cells. Labelling of tolDC with ^19^F-NP did not change the number of cells expressing these markers, although the expression levels of both markers were reduced. Importantly, however, the markers used to define the ‘tolerogenic characteristics’ of tolDC (maturation marker CD83 and the co-stimulatory molecule CD86) were not increased in ^19^F-tolDC. In fact, the percentage of CD86 positive cells was even lower in ^19^F-tolDC as compared to unlabelled tolDC. TLR2 expression was determined because it is a known dexamethasone-induced marker which is expressed by tolDC (7). Although it is not part of the formal QC release criteria of our previous clinical-grade tolDC product, it has been included as a ‘for information only’ QC marker. Although expression levels of TLR2 were reduced in ^19^F-tolDC (**Figure 2**), they remained higher than levels expressed by matDC for each individual product (data not shown). Not surprisingly, contamination of the tolDC product with other cell types was not affected by labelling with ^19^F-NP. The data demonstrate that, with regard to these established QC release markers, the impact of labelling with ^19^F-NP on tolDC is marginal and acceptable, particularly because the particles did not reverse the Dex/VitD3-induced inhibition of DC maturation.

### ^19^F-tolDC retain an anti-inflammatory cytokine profile

**Figure 3:**
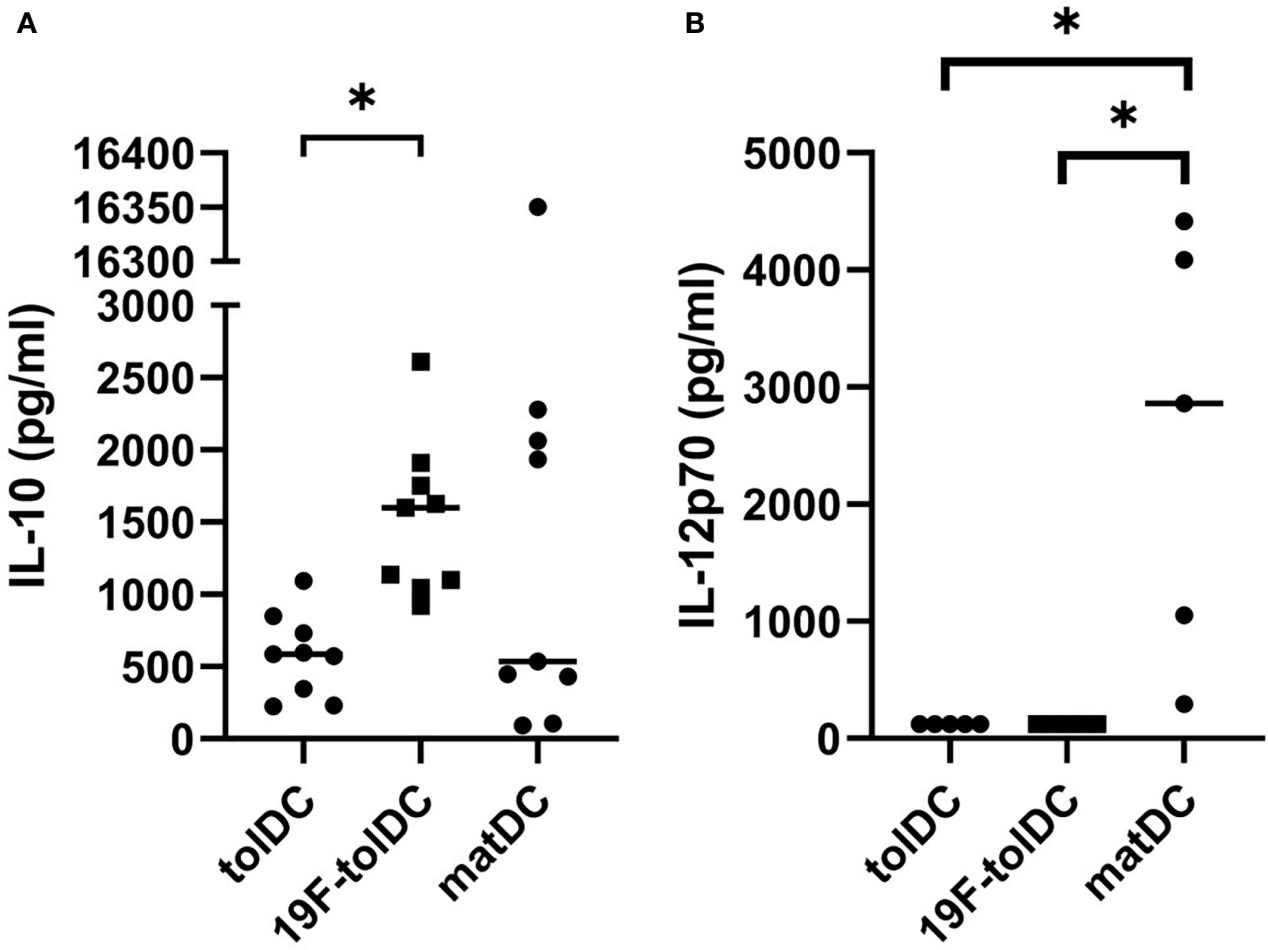
Cytokine secretion by tolDC and ^19^F-tolDC. ^19^F-NP (2.2 mg/ml) was added to tolDC for the last 20-24 hours of culture, after which cell-free supernatant were harvested and frozen at -80°C. Supernatants were thawed and levels of the QC cytokines IL-10 **(A)** and IL-12p70 **(B)** were assessed by ELISA. Graphs show paired experiments with a line indicating the median (*p≤ 0.05).

We assessed whether labelling of tolDC with ^19^F-NP affected their cytokine secretion profile. We have previously shown that tolDC are characterised by production of anti-inflammatory IL-10 and low or undetectable levels of pro-inflammatory IL-12p70 (5–7), and both cytokines are part of the tolDC QC release criteria. ^19^F-tolDC were found to secrete significantly more IL-10 than unlabelled tolDC (**Figure 3A**) whilst, importantly, production of IL-12p70 was not enhanced (**Figure 3B**). These data support the notion that labelling of tolDC with ^19^F-NP does not negatively affect their tolerogenic features.

### ^19^F-tolDC do not induce IFN-γ expression in allogeneic PBMC

**Figure 4:**
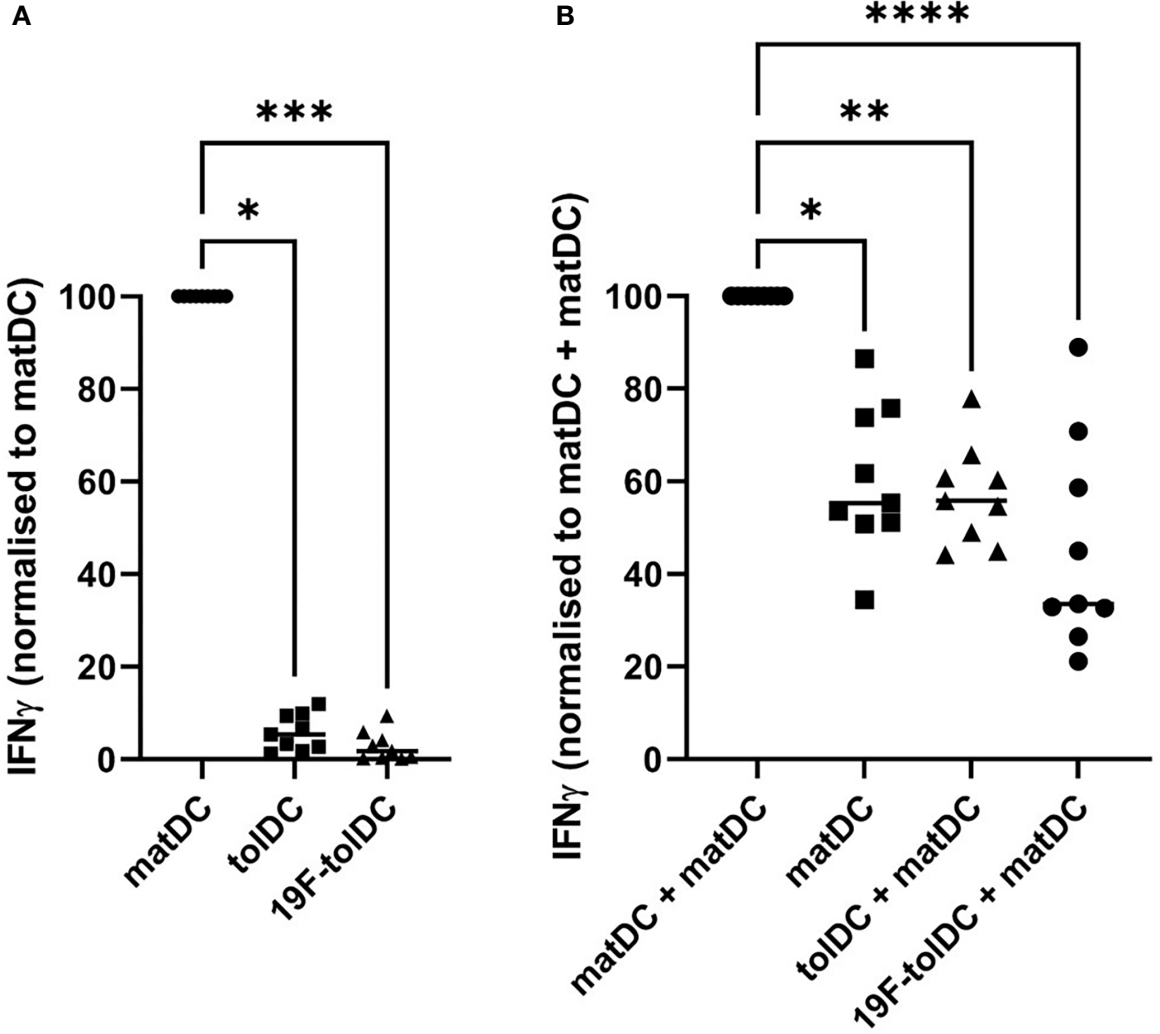
Comparison of IFNγ induction by tolDC and ^19^F-tolDC in allogeneic PBMC. Frozen PBMC gathered from 4 different donors allogeneic to the DC donor were thawed, counted and plated out at 10^5^ cells per well in a 96-well plate. Individual and combinations of fresh DC were added at 10^4^ per condition. Supernatants were collected after 4 days. Levels of IFNγ were determined by ELISA. **(A)** Values were normalised by dividing the IFNγ values for the tolDC and ^19^F-tolDC by the value for matDC then multiplying by 100. **(B)** Co-cultures of various DC combinations and allogeneic PBMC. Values were normalised by dividing the values for matDC, tolDC + matDC and ^19^F-tolDC + matDC by the value for matDC + matDC then multiplying by 100. Graphs contain paired experiments with the median value represented by a line (*p≤ 0.05, **p ≤ 0.01,***p ≤0.001, ****p ≤0.0001). Supernatants from PBMC alone controls were included in these experiments in which very low to undetectable levels of IFNγ were determined.

Current QC testing of Advanced Therapy Medicinal Products needs to include a quantitative measurement of a relevant biological activity, also referred to as a ‘potency assay’. Because a functional hallmark of tolDC is their reduced ability to induce IFN-γ in allogeneic T cells (5–7), we standardised a Good Manufacturing Practice (GMP)-compliant, short *in vitro* assay in which the ability of tolDC and matDC to induce IFN-γ production in allogeneic PBMC is compared. As shown in **Figure 4A**, both unlabelled and ^19^F-NP-labelled tolDC had similarly reduced ability to induce IFN-γ production in this assay when compared to matDC. In addition, further co-culture experiments showed that ^19^F-NP-labelled tolDC did not enhance the ability of matDC to induce IFN-γ (**Figure 4B**). Together with the phenotypic and cytokine secretion data, these functional experiments show that ^19^F-NP is compatible with tolDC.

## Discussion

This study used a set of validated QC criteria developed for our clinical trials with tolDC to investigate whether ^19^F-NP are suitable for labelling of these cells for ^19^F-MRI. We established that ^19^F-NP at a concentration of 2.2 mg/ml had measurable but acceptable effects on tolDC viability, yield and phenotype. The lower expression of HLA-DR on ^19^F-tolDC was noted but not deemed to constitute a problem, since lower expression of HLA-DR has been reported for a number of tolDC types without affecting their ability to regulate the immune response (22). Importantly, labelling of tolDC with ^19^F-NP did not revert these cells into immunogenic, mature DC. The latter was confirmed by a lack of enhanced i) expression of typical mature DC surface markers (CD83, CD86), ii) secretion of IL-12p70 and iii) capacity to induce IFN-γ in allogeneic CD4^+^ T cells. Although others have reported that uptake of ^19^F-particles can enhance DC maturation, this was shown to be dependent on the size of the particles, with only particles >500 nm having this effect (17). The smaller size of the ^19^F-NP nanoparticles (appr. 200 nm) may therefore explain the lack of DC maturation. In addition, we have previously shown that tolDC are maturation-resistant, even when exposed to Toll-like receptor agonists such as LPS or peptidoglycan (7).

We found that virtually all tolDC efficiently ingested ^19^F-NP, which was anticipated as tolDC are monocyte-derived DC, and this type of DC has excellent endocytic capacity that is comparable to monocyte-derived- and tissue-macrophages (23). In addition, the main tolerising agent dexamethasone used here to generate tolDC has been reported to enhance the endocytic activity of monocyte-derived DC (24) However, we did note a weak negative relationship between the ^19^F-NP dose and cell viability, suggesting that excessive endocytosis of these nanoparticles may lead to cell death. Careful dose optimisation is therefore required to ensure maximum detection by MRI without cell overloading-related toxicity. We also noted that endocytosis of ^19^F-NP led to the formation of large endocytic vesicles in which individual nanoparticles could not be discerned, and that the ICG had leaked out into the cytoplasm. This suggests that these nanoparticles disassemble intracellularly and release their cargo. Whether the large vesicles interfere with the intracellular trafficking of molecules is unknown and would require further investigation; however, it should be noted that the secretion of the key immunoregulatory cytokine IL-10 was unaffected.

Although there was some variability in the MRI signal-to-noise ratio (SNR) between batches of ^19^F-tolDC products, it is estimated that the SNRs obtained using ^19^F-NP at 2.2 mg/ml at 3T present a limit of detection of appr. 150,000 tolDC using the ^19^F RF coil and scan parameters employed in our study where a minimum distinguishable signal is defined as a SNR over 5. This would be more than sufficient for our planned tolDC clinical trial, for which the tolDC dose will be 10 million cells. However, the following caveats need to be taken into consideration, which may increase the limit of detection *in vivo*. Firstly, the location of tolDC will play a role in visibility. Whilst an MRI scanner equipped with a ^19^F surface coil can detect ^19^F-tolDC located in tissues close to the skin (e.g. dermis, superficial lymph nodes), coil sensitivity decreases with distance and thus detection of labelled cells in tissues deeper in the body may present more of a challenge. Furthermore, detection will be dependent on the dispersal of the tolDC after injection, either local dispersal that will have a negative impact on the MRI signal, and also migration of tolDC to other locations. Indeed, Ahrens et al. (19) have shown that 24 hour after intradermal injection, detection of ^19^F-labelled human mature DC had decreased by 50%, indicating that the cells had migrated from the injection site, for example to the draining lymph node, or had died and subsequently had been cleared by tissue macrophages. Regardless, no ^19^F signal could be detected in locations other than the original injection site. It is therefore unlikely that the secondary location of ^19^F-tolDC after migration from the primary injection site can be determined by ^19^F-MRI unless a substantial density of labelled cells is achieved at a destination location. Despite this limitation, there remains real value in being able to determine the rate of ‘cell efflux’ from the injection site, which could be through cell migration and/or, considering the short lifespan of tolDC, through clearance of dead tolDC. Further value lies in the ability to verify that cells have been injected correctly. For example, intranodal administration of DC can be hampered by incorrect injection and/or undesired backflow of the cells. Indeed, de Vries et al. (25) showed that correct injection of DC in regional lymph nodes under ultrasound guidance was only successful in ~50% of cases, possibly explaining variable immunological and clinical responses in treated patients.

In conclusion, we have shown that labelling of therapeutic tolDC with ^19^F-NP is feasible and would allow for detection of these cells at the injection site by MRI. Information about the accuracy of tolDC injection as well as their rate of efflux would provide additional novel information about the *in vivo* dynamics of this cell therapy, which could potentially increase our understanding of its clinical effectiveness. Further approaches to improve the sensitivity of ^19^F-MRI would be needed for tracking tolDC migration to secondary sites; these could include imaging at higher clinical field strengths, improvement of hardware/coils or optimisation of imaging pulse sequences (14). Finally, although this study did not identify an impact of ^19^F-NP labelling sufficient to interfere with critical QC or release criteria of tolDC, it cannot be excluded that the labelling process did affect some aspects of tolDC function. Ultimately, label-free imaging of tolDC would be the ideal approach, and recently some headway has been made by applying chemical exchange saturation transfer MRI for the tracking of mesenchymal stem cells (26).

## Data availability statement

The original contributions presented in the study are included in the article/**Supplementary Material**. Further inquiries can be directed to the corresponding author.

## Ethics statement

The studies involving human participants were reviewed and approved by The Animal Welfare and Ethical Review Body(AWERB), Newcastle University. The patients/participants provided their written informed consent to participate in this study. The data presented in **Supplementary Figure 1** were obtained as part of the AuToDeCRA trial. This trial was approved by the Medicines and Healthcare Products Regulatory Agency and by the National Research Ethics Service Committee North East (Sunderland). The trial was conducted according to the International Council for Harmonisation of Technical Requirements for Pharmaceuticals for Human Use Good Clinical Practice (ICH GCP) and the Declaration of Helsinki.

## Author contributions

FC carried out experimental work, analysed data, drafted the Figures and drafted the manuscript; MN carried out the MRI work, analysed data, contributed to drafting the manuscript, provided expert advice on MRI; MW carried out the preliminary experiments that informed study design; IdV provided the ^19^F-NPs and expert advice on ^19^F-NPs; AEA, JD, and AP provided expert help and advice on tolDC cultures and functional assays, and contributed to the ^111^Indium labelling study; JS and IN provided expert help and advice on tolDC cultures and functional assays; NM, DH, DC, and AD provided expert advice on GMP and QC requirements; AB and PT provided expert advice on MRI; JI provided expert advice and led the ^111^Indium labelling study; CH conceived, designed and led the study, wrote the manuscript. All authors read, edited and approved the final manuscript.

## Funding

This work has received funding from Versus Arthritis grant no 21811, the Innovative Medicines Initiative 2 Joint Undertaking under grant agreement no 777357, and the European Union’s Horizon 2020 research and innovation programme under grant agreement No 860003. The NIHR Newcastle Biomedical Research Centre (BRC) is a partnership between Newcastle Hospitals NHS Foundation Trust and Newcastle University, funded by the National Institute for Health and Care Research (NIHR). This paper presents independent research supported by the NIHR Newcastle Biomedical Research Centre (BRC). JI is a NIHR Senior Investigator. The views expressed are those of the author(s) and not necessarily those of the NIHR or the Department of Health and Social Care.

## Acknowledgments

We thank the Newcastle University Flow Cytometry Core Facility (FCCF) for assistance. The authors thank Dr. David Bulmer of the Newcastle University BioImaging Unit for their support & assistance in confocal microscopy work. We acknowledge the Newcastle University EM Research Services for assistance with the generation of EM images (BBSRC grant reference BB/R013942/1). We thank Prof. Dr. Eva Martinez-Caceres (Germans Trias i Pujol University Hospital and Research Institute) for helpful advice on the potency assay.

## Conflict of interest

The authors declare that the research was conducted in the absence of any commercial or financial relationships that could be construed as a potential conflict of interest.

The reviewer PR declared participation with the authors in the INSTRuCT Consortium to the handling editor.

## Publisher’s note

All claims expressed in this article are solely those of the authors and do not necessarily represent those of their affiliated organizations, or those of the publisher, the editors and the reviewers. Any product that may be evaluated in this article, or claim that may be made by its manufacturer, is not guaranteed or endorsed by the publisher.
