# Supplementary material for: Fluorine labelling of therapeutic human tolerogenic dendritic cells for 19F-magnetic resonance imaging
Source: Front Immunol. 2022 Oct 3;13:988667. doi: 10.3389/fimmu.2022.988667 (PMC9574244; doi:10.3389/fimmu.2022.988667)
Supplement: Supplementary file 1 [file DataSheet_1.pdf]

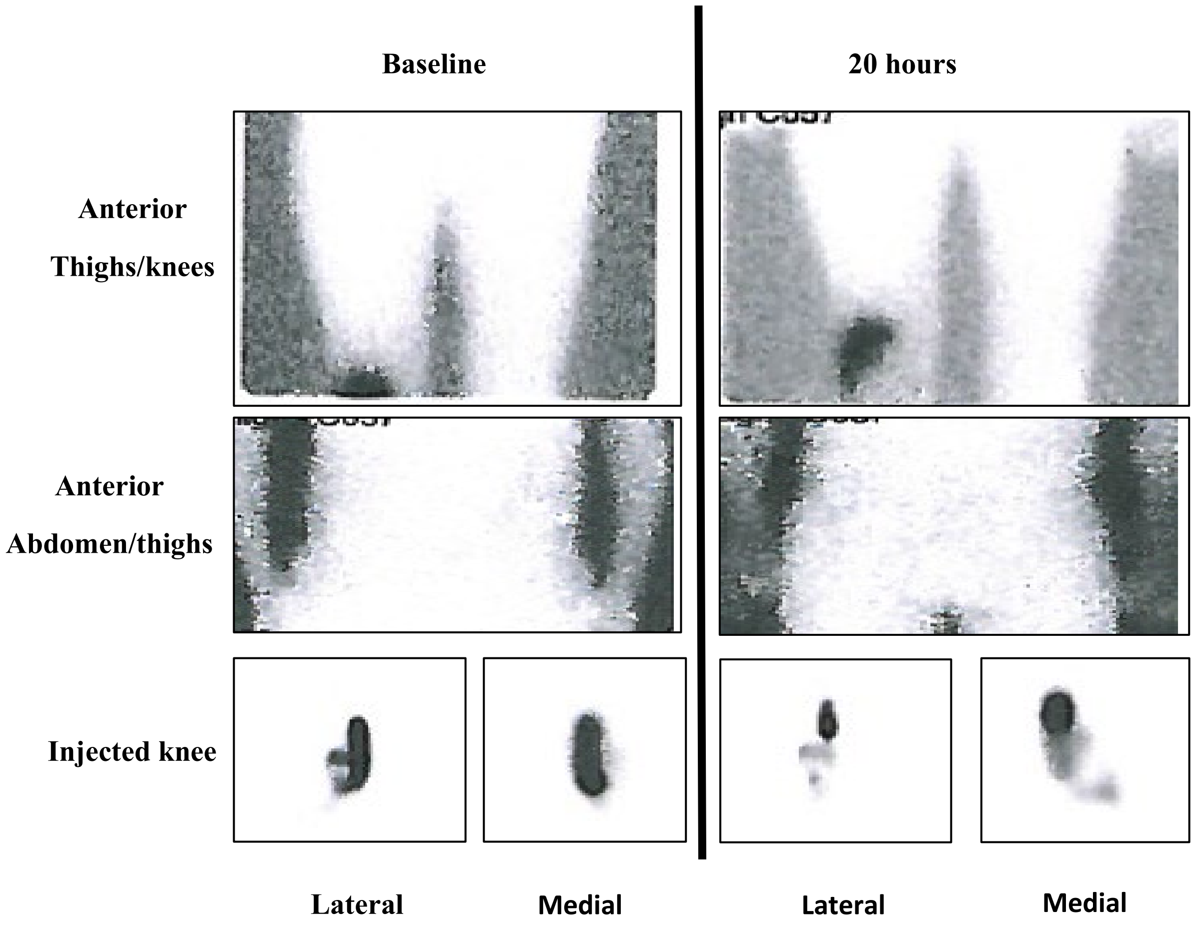

Supplementary Figure 1. Images taken at baseline and 20 hours after injection of 10 x 10^6 ^{111}Indium labelled tolDC into an inflamed knee. The knee signal decays after 20 hours but there is no appearance of signal in inguinal lymph nodes. *^{111}Indium labelling of tolDC:* tolDC were resuspended to 10x10^6 cells/ml in saline containing 0.1 % autologous plasma. 30 MBq of ^{111}Indium (In) chloride (Mallinckrodt Inc.) in tropolone (0.054 % w/v in saline buffer; Oxford Pharmacy Stores) was added before incubation for 10 minutes at room temperature. tolDCs were washed once in saline followed by a second wash in saline containing 50 % autologous plasma before resuspension of the cells in PBS containing 1 % human serum albumin. Labelling efficiency was determined by counting the activity in the wash supernatant and cells. The ^{111}In-labelled cells were injected into the knee joint by arthroscopy and gamma camera imaging was performed at 1 hour and 20 hours post-dose administration in Nuclear Medicine, Royal Victoria Infirmary, Newcastle upon Tyne.
