# Supplementary material for: Fluorine labelling of therapeutic human tolerogenic dendritic cells for 19F-magnetic resonance imaging
Source: Front Immunol. 2022 Oct 3;13:988667. doi: 10.3389/fimmu.2022.988667 (PMC9574244; doi:10.3389/fimmu.2022.988667)
Supplement: Supplementary file 2 [file DataSheet_2.pdf]

**Target antigen**	**Manufacturer**	**Product number**
HLA-DR	Biolegend	307636
CD11c	Biolegend	371511
CD83	BD	561959
CD86	BD	557344
TLR2	Thermo Scientific	12-9922-41

Supplementary Table 1. A list of the anti-human monoclonal antibodies used to assess the phenotype of DC.
